# RNA expression differences in prostate tumors and tumor-adjacent stroma between Black and White Americans

**DOI:** 10.18632/oncotarget.28024

**Published:** 2021-07-20

**Authors:** Farah Rahmatpanah, Gabriela De Robles, Michael Lilly, Thomas Keane, Vinay Kumar, Dan Mercola, Pavneet Randhawa, Michael McClelland

**Affiliations:** ^1^Department of Pathology and Laboratory Medicine, University of California, Irvine, CA 92697, USA; ^2^Department of Hematology and Oncology, Medical University of South Carolina, Charleston, SC 29425, USA; ^3^Department of Urology, Medical University of South Carolina, Charleston, SC 29425, USA; ^4^Department of Microbiology and Molecular Genetics, University of California, Irvine, CA 92697, USA

**Keywords:** prostate cancer, tumor-adjacent stroma, African ancestry, European ancestry, RNA-seq analysis

## Abstract

Prostate cancer (PCa) in Black Americans (BA) is diagnosed at an earlier median age and a more advanced stage than PCa in White Americans (WA). Tumor-adjacent stroma (TAS) plays a critical role in tumorigenesis of prostate cancer. We examined RNA expression in both tumor and TAS of BA compared to WA. After evaluating the geographical ancestry of each sample, preliminary analysis of our own RNA-seq data of 7 BA and 7 WA TAS revealed 1706 downregulated and 1844 upregulated genes in BA relative to WA PCa patients (*p*_adj_ < 0.05). An assessment of published RNA-seq data of clinically matched tumor-enriched tissues from 15 BA and 30 WA patients revealed 932 upregulated and 476 downregulated genes in BA relative to WA (*p*_adj_ < 0.05). When TAS and tumor epithelial cohorts were compared for the top 2500 statistically significant genes, immune responses were downregulated in BA vs WA TAS, while T cell-exhaustion pathways and the immune checkpoint gene CTLA4 were upregulated in BA vs WA tumors. We found fewer activated dendritic cells in tumor and more CD8 T-cells in TAS of BA versus WA PCa patients. Further characterization of these differences in the immune response of PCa patients of distinct geographical ancestry could help to improve diagnostics, prognostics, and therapy.

## INTRODUCTION

Prostate cancer (PCa) of Black Americans (BA) is diagnosed at an earlier median age and more advanced stage than PCa of White American patients (WA), and has a poorer prognosis and significantly higher mortality rate. The differences in mortality persist even after accounting for socioeconomic and environmental factors [1]. Recent studies indicate that 35% of American PCa patients of African descent assigned to active surveillance gradually undergo aggressive treatments within 5 years due to disease progression, compared to 15% of American patients of European (Caucasian) descent afflicted with the disease [2–4].

The mechanism of how race contributes to aggressive PCa in BA patients is not well understood [5]. However, there is evidence for genetic polymorphisms, differences in gene expression, and differential DNA methylation between PCa patients of distinct racial descent [6–8].

Tumor-adjacent stromal cells (TAS) play a critical role in tumorigenesis of PCa [9, 10]. Several stimulatory paracrine growth factors that act on epithelial cells are produced by stroma, including DHT, PDGF, IGF1, VEGF and EGF [11], suggesting that the TAS of BA patients may have properties that promote the more aggressive phenotype of the disease. Studies from our group have indicated that TAS of PCa patients has hundreds of significant RNA expression changes compared to normal stroma tissue [9]. We have exploited these findings to develop biomarker panels that reliably distinguish normal prostates from tumor-bearing prostates or distinguish good disease outcomes from poor disease outcomes (prognosis) for individual patients [9, 12]. These observations illustrate the potential of stroma characterization to assist in the management of prostate cancer [13]. Using fresh frozen tissues and microarray technique we found that the TAS of BA patients has many down-regulated genes relative to WA patients and that many of these genes encoded proteins with functions associated with immune response [14].

The primary aim of this study was to uncover RNA expression differences in tumor and in TAS of BA versus WA patients, since some of these differences might contribute to the observed higher rate of aggressive PCa. To that end, we analyzed publicly available RNA-seq data (GEO database GSE54460) from 99 PCa tumor-enriched formalin-fixed, paraffin-embedded (FFPE) prostatectomy samples [15]. After matching for age, Gleason score, relapse status, and ancestry composition, a total of 45 PCa samples from the Atlanta VA Medical Center (15 BA and 30 WA) met the criteria for further analysis. Subsequently, we performed RNA-seq on TAS of 9 BA and 11 WA PCa patients using FFPE tissues obtained at the University of California, Irvine, and the Medical University of South Carolina. We determined differences in RNA expression of tumor-enriched and TAS PCa samples from BA versus WA patients. The significantly different pathways between PCa patients of distinct ancestries included immunity response mechanisms worthy of further exploration.

## RESULTS

### Confirmation of self-reported ancestry using LASER analysis

**Figure 1:**
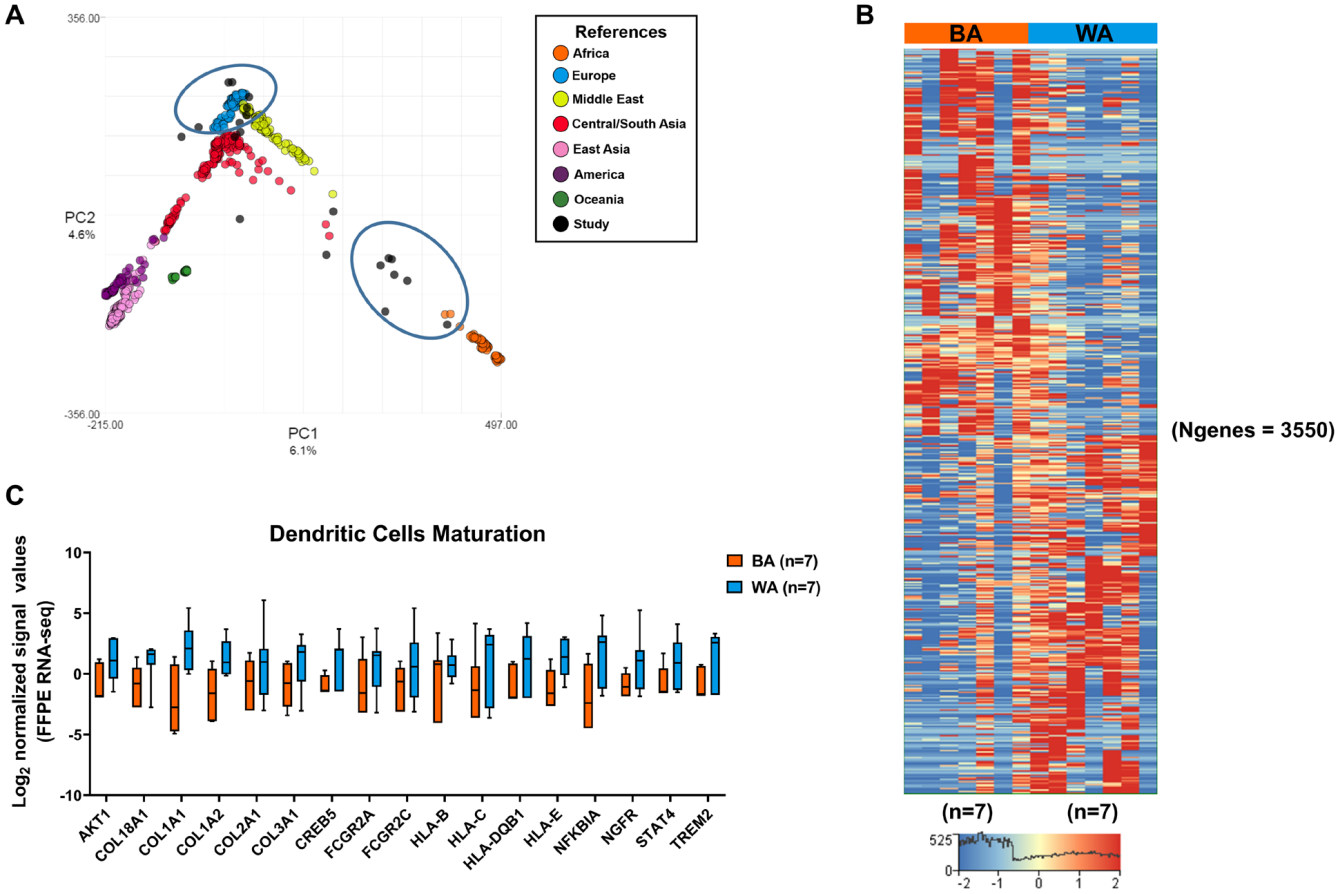
Differential gene expression in tumor-adjacent stroma (TAS) of Black American and White American PCa patients. (**A**) Patient ancestry was determined using LASER (see Materials and Methods). Samples sequenced in our study are indicated in black, those used in our comparisons are encircled. (**B**) Hierarchical analysis of genes that are differentially expressed in TAS of BA (*n* = 7) versus WA (*n* = 7) PCa patients (*p*_adj_ < 0.05 and ± 2.5-fold change). Red depicts up- and blue downregulation. Clustered based on genes. N is the number of genes. (**C**) Overrepresentation of dendritic cell maturation pathways among significantly downregulated genes in the TAS of BA (*n* = 7) compared to WA (*n* = 7) prostate cancer patients. Y-axis represents normalized log_2_ signal values (normalized reads) (*p*_adj_ < 0.05).

**Figure 2:**
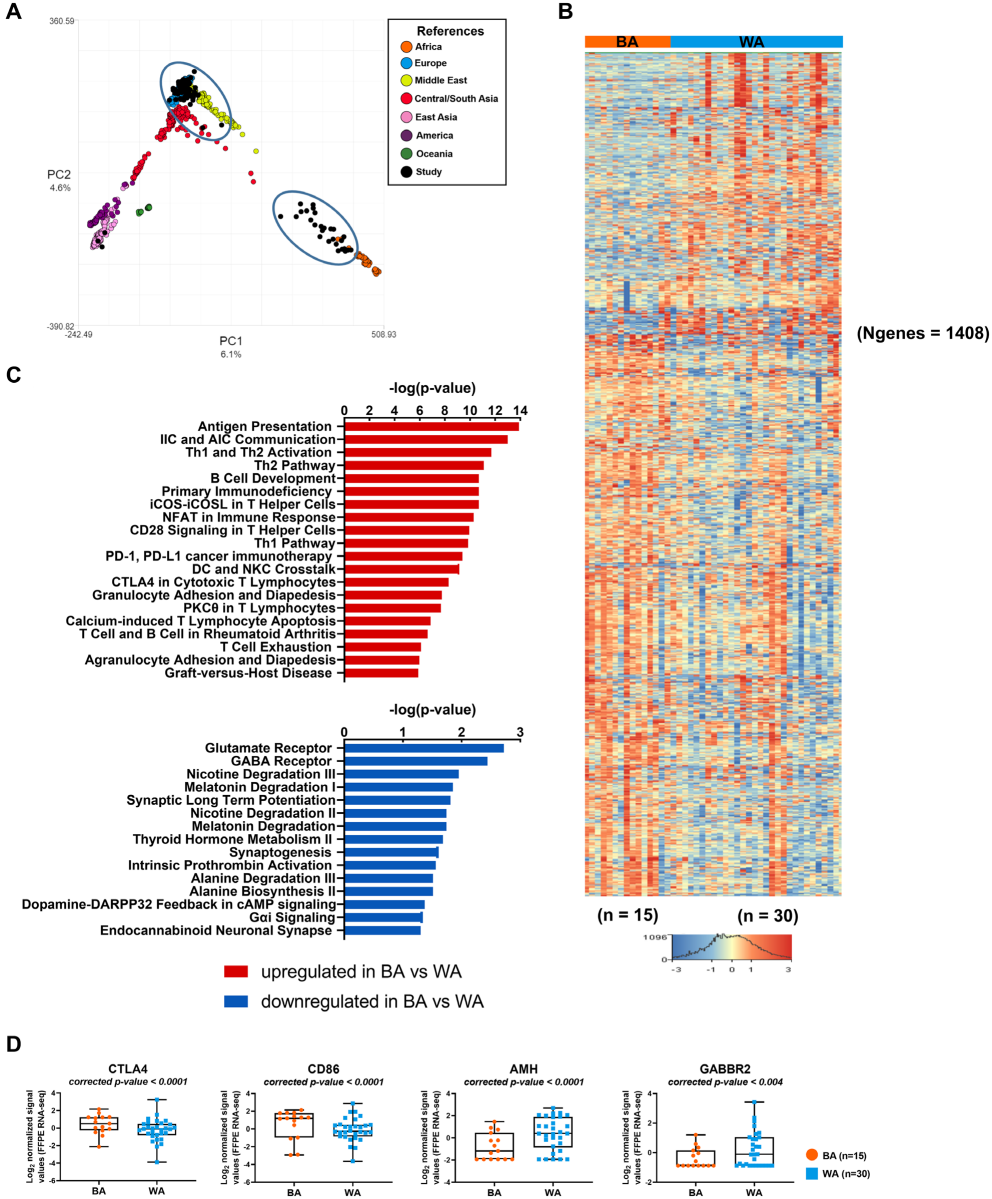
Transcriptome analysis of tumor samples of PCa patients with different geographical ancestries. (**A**) Sample ancestry of 99 published PCa FFPE tissue samples (GSE54460) was determined using LASER (see Materials and Methods). Samples used in our comparisons are black and encircled. (**B**) Differential gene expression changes in clinically matched BA (*n* = 15) compared to WA (*n* = 30) prostate tumor samples (*p*_adj_ < 0.05 and ± 1.5-fold change). Red depicts up- and blue depicts downregulation. Clustered based on genes. *N* = number of genes. (**C**) Top 20 canonical pathways of upregulated differentially transcribed genes and top 15 canonical pathways of downregulated differentially transcribed genes in BA (*n* = 15) compared to WA (*n* = 30) prostate tumor samples, after Ingenuity Pathway Analysis (-log_10_ *p* value ≥ 1.30). (**D**) Scatter plots of differentially expressed immune checkpoint inhibitor genes and AMH (*p*_adj_ < 0.05 and ± 1.5-fold change) in BA (*n* = 15) versus WA (*n* = 30) PCa samples.

For all samples analyzed in this study, we used *Locating Ancestry from Sequence Reads* (LASER) software to determine the accuracy of the self-reported race [16, 17], to assign ancestry to samples from patients lacking this information [18], and to estimate the ancestry composition for each individual (Figures 1A, 2A, and Supplementary Tables 1–2).

We extracted RNA from dissected FFPE tumor-adjacent stroma of nine self-identified black and eleven self-identified white patients matched for clinical parameters. Of nine self-identified black patients, seven clustered with African (prominent ancestry) and two with Middle Eastern ancestry. Among eleven self-identified white patients, five had patterns matching a prominent European descent, two patients clustered with Middle Eastern reference samples, whereas the rest of the patients clustered with reference samples of Asian ancestry (Table 1). Patients that were not confirmed by LASER to be of the expected geographical ancestry were excluded from further analysis. This applied to the two self-identified black patients with predominantly Middle Eastern ancestry and to the four self-identified white patients with Asian ancestry.

**Table 1:** Individual ancestry estimation of 99 FFPE PCa tumor-enriched samples by analysis of RNA sequence reads using LASER

Self-identified race	No. of patients	Ancestry composition according to LASER		Geographical ancestries (LASER)
		100%	< 100%	
**Black**	22	15 A	7 (A)	22 African
**White**	46	30 E 5 ME 1 CSA	10 (9 E, 1 CSA)	39 European 5 Middle Eastern 2 Central/South Asian
**Unassigned**	31	17 E 5 A 2 ME 2 CSA	5 (3 E, 2 E/ME)	20 European 5 African 2 Middle Eastern 2 Central/South Asian 2 European/Middle Eastern

Samples of mixed ancestry are assigned a category based on their most prominent ancestry (70%). Two samples were identified as equal mixtures of European and Middle Eastern ancestry. Samples were obtained from the *Gene Expression Omnibus* database (GSE54460). Abbreviations: E: European ancestry; A: African ancestry; ME: Middle Eastern ancestry; CSA: Central/South Asian ancestry.

We also obtained RNA-seq and clinical data derived from FFPE prostate cancer tumor-enriched samples of 99 patients [15], reported in the Gene Expression Omnibus database as GSE54460. Of the 46 self-identifying White patients, 30 clustered with European references (65%), five with Middle Eastern references (11%), and one with Asian ancestry that was eliminated from further analysis. The remaining 10 self-identifying white patients had admixed ancestry DNA compositions (Figure 2A, Supplementary Figure 1, Table 1, Supplementary Table 2). A total of 15 of the 22 self-identifying Black patients had DNA that matched African ancestry, while the other 7 patients had admixed ancestry composition. Of the 31 PCa patients lacking geographical ancestry information, LASER assigned 5 to African, 24 to European, and 2 to Asian ancestry. Note that the estimates of ancestry using LASER are approximate and rely on the distance to the nearest member of each of the 51 geographical groups. Consequently, when the match with one group approaches 100%, the match tends to be exaggerated because of an insufficient connection to other ancestries.

We selected both Black and White PCa patients from the Atlanta VA Medical Center with similar ancestral compositions for comparative analysis [15]. Previous population studies using 1000 genomes chosen for their geographical diversity revealed a smaller genetic distance between Middle Eastern and European compared to African individuals [16, 17]. Therefore, we grouped patients of European and Middle Eastern ancestry in this dataset. Of the 99 samples, 15 BA patients were matched to 30 WA patients based on clinical characteristics, including age, follow-up time (months), Gleason score, and biochemical relapse (Supplementary Table 3).

### RNA-seq and pathway analysis of TAS of BA and WA PCa patients

We used the Truseq RNA Access method of Illumina to investigate the gene expression differences in TAS of BA (*n* = 9) and WA (*n* = 11) prostate cancer patients (Supplementary Table 1). A total of six patients (two BA and four WA) that were not confirmed by LASER to be of the expected geographical ancestry were excluded from further analysis. For the TAS cohort, BA and WA patients were matched for age (average age in both cohorts was 61 years), follow-up time (average follow-up time was 37 months for BA and 44 months for WA patients), and cases of biochemical relapse (six in BA, nine in WA). BA patients have more aggressive tumors at every clinical stage of the disease, resulting in poorer prognosis and increased mortality [14]. To mitigate the variation in the number of sequencing mapped reads among TAS samples (Supplementary Table 1), we used the DESeq normalization, which uses the median of ratios method, to account for differences in sequencing reads (i.e., depth) [19, 20]. RNA sequencing of tumor-adjacent stroma from PCa FFPE tissues identified 1844 significantly upregulated and 1706 significantly downregulated genes in TAS of BA (*n* = 7) as compared to WA (*n* = 7) PCa patients (*p*_adj_ < 0.05 and |FC| > 2.5) (Figure 1B, Supplementary Dataset 1; Supplementary Table 1A). A total of 6 patients that were not confirmed by LASER to be of the expected geographical ancestry were excluded from further analysis.

The core pathway analysis in the Ingenuity Pathway Analysis (IPA) package (QIAGEN Inc, USA) and the pathway analysis module in Strand NGS 3.1 were used to identify the most significant pathways (*p* < 0.05) that distinguish TAS of BA PCa patients from those of WA PCa patients in our sample set (Figure 1C, Supplementary Figure 2, Supplementary Dataset 1; Supplementary Table 1B–1C). Pathways significantly enriched in genes with reduced RNA expression in BA TAS included immune responses, such as natural killer cell signaling, B-cell receptor signaling, dendritic cell maturation, IL-6 signaling, oncostatin M signaling, the antigen presentation pathways and mTOR signaling (-log_10_ *p* value = 3.16, 1.86, 1.74, 1.79, 1.69, 1.46, 2.18, respectively). The significantly upregulated genes in TAS of BA versus White PCa patients were enriched in metabolic pathways including phosphoribosyl pyrophosphate synthetase (PRPP) biosynthesis I (-log_10_ *p* value = 1.93), triacylglycerol degradation (-log_10_ *p* value = 1.45) and pyrimidine deoxyribonucleotides *de novo* biosynthesis I (-log_10_ *p* value = 1.32). Additionally, genes involved in tRNA charging, IL-12 signaling in production of macrophages and bone morphogenetic protein (BMP) signaling pathways were overrepresented with -log_10_ *p* values of 2.63, 1.42, and 1.42, respectively.

### Gene expression profiles of tumor-enriched samples from PCa patients

To investigate the influence of genetic ancestry on gene expression among tumor-enriched samples from PCa patients, we examined publicly available data [15] from self-identified Black patients that were at least of 80% African ancestry and self-identified White patients that were at least of 80% European or Middle Eastern ancestry. Patients were selected to be clinically matched based on their Gleason score, age, and biochemical relapse status. Among samples from 99 patients, we selected 15 BA and 30 WA PCa samples (listed first in Supplementary Table 2). These tumor samples were compared for gene expression differences using RNA-seq data obtained from GSE54460 [15]. 1408 genes were statistically significantly differentially expressed (FDR < 0.05 and FC ≥ ±1.5) in BA patients as compared to WA (932 up- and 476 down-regulated genes) (Figure 2B, Supplementary Dataset 2; Supplementary Table 2A). The core pathway analysis in the IPA tool was used to identify the most significantly dysregulated biological functions (-log_10_ *p* > 1.3, which is equivalent to *p* < 0.05) associated with up- and down-regulated genes in BA vs WA PCa patients (Figure 2C, Supplementary Dataset 2, Supplementary Table 2B–2C). Among the top 20 pathways (-log_10_ *p* > 5.87) associated with upregulated genes in BA versus WA patients were immune and inflammatory responses including T cell-exhaustion signaling and CTLA4 signaling in cytotoxic T lymphocytes. Several chemokines, chemokine ligands, cytokines, matrix metallopeptidases (MMPs), integrin and genes of lymphocyte migrations were upregulated in BA patients as compared to WA, including CXCL10, CXCL2, HLA-A, CCL2, CCL21, CCL3, CCR4, CCR5, CD4, ITGA4, ITGB2, ICAM1, ICAM2, CD86, and CTLA4 (Figure 2D, Supplementary Dataset 2; Supplementary Table 2A). Our analysis suggests a much stronger activation of inflammation and immune responses in tumor-enriched PCa samples of BA versus WA patients, consistent with previous reports by others [21, 22]. A previous study on 10 self-identified African American and 17 self-identified European American PCa patients observed 242 upregulated genes in tumor epithelium of African American patients [21] that were linked to inflammatory processes and immune responses at an early stage as compared to European American PCa patients. A total of 65 of those genes were also represented in our identified transcripts with *p*_adj_ < 0.05 and |FC|>1.5, with 98% concordance (Supplementary Figure 3) including CTLA4 and CD86.

Pathway analysis of downregulated genes in BA as compared to WA PCa samples identified 15 significantly downregulated pathways (-log_10_ *p* value ≥ 1.3 or *p* < 0.05) (Figure 2C, Supplementary Dataset 2; Supplementary Table 2C). Among these were several metabolic pathways, including alanine biosynthesis II, alanine degradation III, thyroid hormone metabolism II, and melatonin degradation I. They also included GABA receptor signaling. Furthermore, we found the gene encoding the gamma-aminobutyric acid (GABA) B receptor 2 to be downregulated in BA vs WA PCa tumor samples (FDR < 0.05 and FC = −1.51) (Supplementary Dataset 2, Supplementary Table 2A).

We used the IPA tool to identify specific diseases and networks that were overrepresented among the 1408 significantly differentially expressed genes in tumor samples of BA versus WA PCa patients (Supplementary Dataset 2; Supplementary Table 2D–2G). Of 1408 genes shown in Figure 2B, 434 (291 up- and 143 down regulated) were associated with genes specifically involved in prostate cancer (Supplementary Dataset 2, Supplementary Table 2D–2E). Gene network analysis of downregulated genes in BA vs WA PCa samples identified *cancer, developmental disorder, endocrine system disorders* among the top networks (Supplementary Figure 4, Supplementary Dataset 2; Supplementary Table 2F). One key gene of this network is encoding the anti-Mullerian hormone (AMH), also known as Mullerian -inhibiting substance (Figure 2D, Supplementary Figure 4, Supplementary Dataset 2; Supplementary Table 2F). The protein is a member of the TGFβ family that regulates growth, differentiation, and apoptosis in many cells [23]. AMH regulates the Androgen Receptor (AR)-induced gene expression and growth in PCa cells through an NFκB-dependent but Smad1-independent mechanism [24]. The cancer-inhibitory effect of AMH via prevention of cell cycle progression has been documented [25]. Thus, low expression levels of AMH in BA as compared to WA PCa patients may have consequences for disease progression (Supplementary Dataset 2; Supplementary Table 2G).

### Comparison pathway analysis of genes significantly altered in TAS and tumor of Black versus White PCa patients

The top 2500 statistically significantly differentially expressed genes (*p*_adj_ < 0.05) in tumor samples of BA vs WA patients from the GEO database (GSE54460) [15] and in our TAS samples were compared (Supplementary Dataset 3, Supplementary Table 3A–3B). Unsurprisingly, tumor and TAS patterns were entirely different from each other in both WA and BA PCa patients. We identified 243 genes that were differentially expressed in BA *versus* WA patients in both TAS and tumor. Of these 243 genes, 94 (39%) were differentially expressed in the same direction.

**Figure 3:**
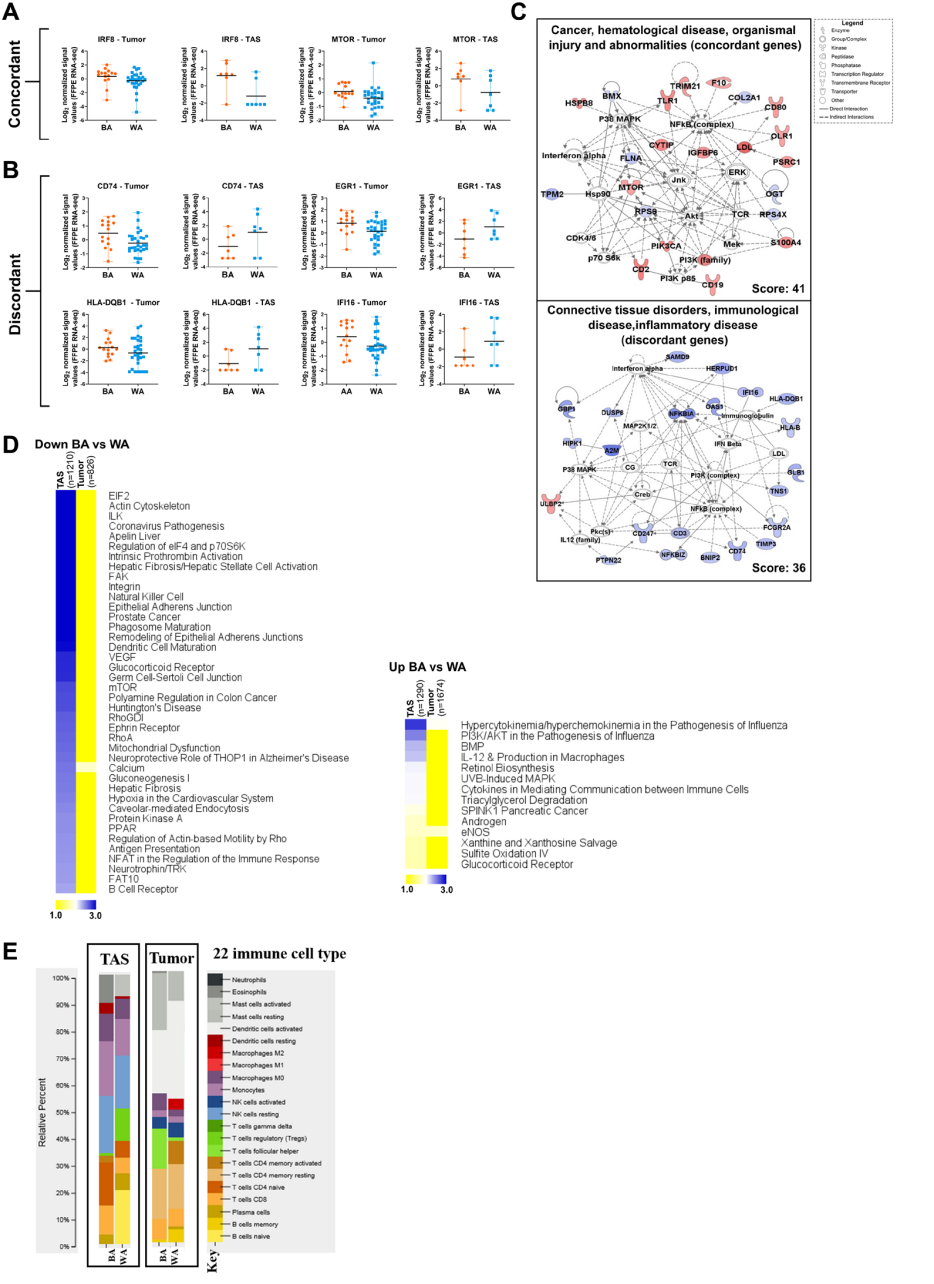
Comparative pathway analysis of significantly differentially expressed genes in BA versus WA PCa patients in tumor and tumor-adjacent stroma (TAS). (**A**–**B**) The top 2500 significantly differentially expressed genes (*p*_adj_ < 0.05) in BA vs WA were compared in TAS and tumor. Notable overlapping genes with the same direction of regulations (concordant) and with different direction of regulation (discordant) in tumor and TAS of BA versus WA PCa patients are shown. Each dot represents a patient. (**C**) Gene network analysis of concordant and discordant genes in tumor and TAS of BA versus WA PCa patients. The networks shown are among those with the highest significance of connections between molecules in the network as indicated by their score. Blue nodes indicate downregulated and red nodes indicate upregulated gene expression in TAS of BA vs WA PCa patients. Darker shades of the nodes indicate larger differential expression ratios. Dotted lines represent indirect interactions while solid lines represent direct interactions. (**D**) Comparative pathway analysis of the top 2500 significantly differentially expressed genes (*p*_adj_ < 0.05) in BA and WA in both tumor and TAS cohorts with concordant and discordant regulations. The top overrepresented signaling pathways (-log_10_ *p* value > 1.30) between TAS and tumor in BA versus WA PCa samples among downregulated (left) and upregulated (right) genes are shown. The heat map is generated from the –log_10_ *p* values, using MeV (http://mev.tm4.org). n is the number of genes. (**E**) CIBERSORT analysis of 22 immune cell types in tumor epithelia of BA (*n* = 15) and WA (*n* = 30) (*p* < 0.05) and TAS of BA (*n* = 7) and WA (*n* = 7) PCa patients.

Of these 94 concordant genes, 35 were downregulated and 59 upregulated in BA vs WA PCa patients. Among overlapping downregulated genes in BA patients versus WA patients in both tumor and TAS are tumor suppressors p16 (CDKN2A), filamin A (FLNA) and ladinin 1 (LAD1). Genes upregulated in both tumor and TAS of BA PCa patients as compared to WA included cell cycle markers CD19, CD2, CD53 and CD80, interferon response factor 8 (IRF8), phosphoinositide 3-kinase (PIK3CA), mechanistic target of rapamycin (mTOR), and metastasis-associated protein S100A4 (Figure 3A, Figure 3C, Supplementary Figure 5, Supplementary Dataset 3; Supplementary Table 3A–3B).

Our analysis also identified 149 genes (61%) that were regulated in BA versus WA in both tumor and TAS, but in the opposite direction. These differences included several genes of antiviral immune response pathways such as HLA-B, HLA-C, HLA-DQB1 IFI16, IFITM3, CD74 (MHC class II transporter), tumor suppressor genes EGF-containing fibulin extracellular matrix protein (EFEMP1), and the transcription factor early growth response 1 (EGR1), all upregulated in BA tumor versus WA tumor, and downregulated in BA TAS versus WA TAS. Additionally, several other genes associated with poor prognosis in other cancers were upregulated in BA TAS but downregulated in BA tumor versus their counterparts in WA PCa patients. These included the oncogenes SRY-box transcription factor 4 (SOX4) [26] and ubiquitin-specific peptidase 6 (USP6) [27] (Supplementary Dataset 3; Supplementary Table 3A–3B).

We performed a comparative pathways analysis on the top 2500 significantly differentially expressed genes (*p*_adj_ < 0.05) that distinguished tumors from BA vs WA patients (1674 up- and 826 down-regulated) and a similar analysis for the top 2500 genes for TAS (1290 up- and 1210 downregulated) (Figure 3D, Supplementary Dataset 3; Supplementary Table 3C–3D). Pathways that were significantly downregulated in TAS of BA patients (*p* < 0.05) but not significant in the tumor comparison included antiviral immune response pathways such as dendritic cell maturations, B cell receptor signaling, natural killer cell signaling, mTOR signaling, antigen presentation and regulation of eIF4 and p70S6K signaling (Figure 3D, Supplementary Dataset 3; Supplementary Table 3C). Metabolic pathways enriched among upregulated genes (*p* < 0.05) in TAS of BA vs WA PCa patients but not in tumors include triacylglycerol degradation, xanthine and xanthosine salvage, sulfite oxidation IV and glucocorticoid receptor signaling. Additionally, the pathway defining the role of hypercytokinemia/hyperchemokinemia in the pathogenesis of influenza infections and endothelial nitrous oxide synthetase (eNOS) signaling were enriched in significantly upregulated genes in both tumor and TAS of BA vs WA patients (Figure 3D, Supplementary Dataset 3; Supplementary Table 3D).

### Immune cell-type analysis in tumor and tumor-adjacent stroma of BA and WA PCa patients

We employed the *Cell-type Identification by Estimating Relative Subsets of RNA Transcripts* (CIBERSORT) computational tool [28] to characterize the immune cell-type composition in prostate cancer in both TAS (7 BA and 7 WA) and tumor (GSE54460, 15 BA and 30 WA) samples. In this analysis, we used normalized quantified gene expression values as input to CIBERSORT. The results were expressed as relative fractions normalized to 1 (total leukocyte content).

In TAS, although the *p* value could not reach < 0.05 due to the small sample size, higher fractions of naïve CD4 T-cells (16% vs 6%), CD8 T-cells (11% vs 6%), and monocytes (20% vs 14%) were observed in BA as compared to WA PCa patients. In contrast, naïve B-cells and activating mast cells were present in TAS of WA at 20% and 8%, respectively, but entirely missing in TAS obtained from BA patients. Additionally, resting NK cells were present in both BA and WA TAS of PCa patients at 21% and 20% respectively (Figure 3E).

In tumor-enriched PCa samples, the analysis revealed overall similarities between BA and WA including in the prevalence of activated NK cells and monocytes. However, the BA PCa patients had fewer activated dendritic cells (23% vs 36%, *t*-test *p* value < 0.02) as compared to WA patients (Figure 3E).

## DISCUSSION

One major hurdle in comparisons of tissue samples of differing ancestries is verification of the accuracy of self-identified racial identity, since self-identifying only serves as a moderate to weak proxy for ancestral genotyping [29]. Admixed ancestry and self-identified geographical ancestry might be confounding factors on any cancer health disparity study [30]. Therefore, we used LASER to validate self-identified ancestry, and to assign geographical ancestry based on genetic data to patients without such information in the dataset. Samples from patients that were not confirmed by LASER to be of the expected geographical ancestry were excluded from subsequent analysis.

In our previous studies, we observed that the microenvironment of prostate cancer exhibits hundreds of significant gene expression changes that distinguish TAS from normal and tissues from patients that experience relapse from those that do not [9, 12]. TAS appears to exhibit expression changes in part due to paracrine factors of the tumor, which lead to alterations at a distance from the actual tumor tissue. Additionally, in an analysis of 17 African American and 17 Caucasian American prostate cancer cases using microarray expression data from frozen tissues, we identified altered immune and epithelial to mesenchymal transition (EMT) processes in TAS to play a role in the aggressive nature of PCa in patients of African American ancestry [14]. We now extended these studies to RNA-seq and FFPE PCa tissue blocks, archived pathology tissues with more extensive patient follow-up.

The expression studies we present here are consistent with our prior data and suggest differences in immune response genes in TAS of BA vs WA patients [14]. We find decreased mRNA expression of genes involved in antigen presentation, natural killer cell signaling, dendritic cell maturation, and mTOR-, EIF2- and oncostatin M signaling in BA compared to WA PCa TAS samples. Reduced antigen presentation may result in an immune-tolerant environment that allows tumors to evade host recognition [31]. Oncostatin M signaling, mTOR, and EIF2 all play a critical role in activating the antiviral immune response. Moreover, oncostatin M enhances the expression of type-1 interferon (IFN -β) in response to dsRNA that leads to activation of interferon-stimulated genes in fibroblasts, which contributes to the regulation of cellular immunity [32].

Our data show upregulation of genes in TAS of BA versus WA PCa samples in several metabolic pathways, including genes involved in phosphoribosyl diphosphate biosynthesis (PRPS1, PRPS2). Previous studies suggest an association between PRPS2 and c-Myc-driven cancers [33, 34]. Overexpression of the oncogene c-Myc at both the mRNA and protein level has been reported in patients with biochemically recurrent prostate cancer [35].

To investigate gene expression differences in TAS and tumor epithelium of BA compared to WA PCa patients, we analyzed published RNA-seq data of tumor epithelium from 99 PCa FFPE tissues, obtained from 22 self-identified Black patients, 46 self-identified White patients, and 31 patients with unassigned race/ ethnicity (GSE54460) [15], which we assigned based on LASER results. Patients were matched based on their clinical variables and their ancestry. Unlike expression patterns observed in our TAS samples, gene expression analysis of tumor epithelium revealed upregulated genes in BA versus WA PCa patients in several immune and inflammatory response pathways, including CTLA4 signaling in cytotoxic T cells, T cell exhaustion and PD-1/PD-L1 cancer immunotherapy. Several genes involved in lymphocyte migration were upregulated in tumors of BA versus WA PCa patients, including CCL2, CCL21, CCL3, CCL4, CCR4, CCR5, CD86, and CTLA4. Several of these chemokines, including CCL21, CCL2, and CCL4, recruit anti-tumor leukocytes but can also recruit pro-tumor leukocytes such as Tregs, depending on the type of malignancy [36]. Interaction of CTLA4 with CD86 inhibits T cell activation and is a key negative regulator of the immune response to tumor [37]. A CTLA4 blockade using ipilimumab and tremelimumab prolongs the antitumor immune responses in melanoma and PCa patients [38]. High expression levels of CTLA4 and CD86 in BA patients may therefore be indicative of a dysregulated immune response.

Among key downregulated genes in tumors of BA vs WA PCa samples were the gamma-aminobutyric acid B receptors GABRR2 and GABRR3. Data suggest that the activation of GABBR2 plays an important role in suppressing the proliferation and migration of various human tumor cells and in the inactivation of cAMP-responsive element binding protein (CREB) and extracellular regulated kinase (ERK) in tumor cells [39]. The observed lower expression of these genes may therefore contribute to the aggressive disease in BA PCa patients (Figure 2D).

Comparisons of the top 2500 significantly altered genes (*p*_adj_ < 0.05) in TAS and tumor of BA vs WA PCa patients identified 243 overlapping genes, of which 94 (39%) were concordant. Among the concordant downregulated genes is FLNA, encoding filamin A, which is a regulator of the androgen receptor in prostate cancer [40]. However, it has so far not been investigated in BA PCa patients. Mutation or polymorphisms of the androgen receptor in African American PCa patients are associated with an elevated prostate cancer risk [41]. We did not find differences in expression of this receptor between BA and WA PCa patients; however, our analysis revealed an increased mRNA expression for the androgen receptor in PCa patients with more aggressive disease (i.e., G4+3 vs G3+4), regardless of ancestry (data not shown).

Several important genes emerged in this study that are overexpressed in BA versus WA patients in both tumor and TAS, including PIK3CA, mTOR and CD53. The crosstalk between the PIK3 and mTOR pathways can promote prostate cancer progression [42]. It is noteworthy that we found PIK3CD (one of the three subunits of PIK3 enzyme) to be uniquely upregulated in TAS of BA PCa patients. It has been shown that alternative splicing and overexpression of PIK3CD promote tumor aggressiveness and drug resistance in African American prostate cancer patients [43].

Our data revealed 149 genes (61%) that were differentially but discordantly regulated in BA vs WA PCa patients in both tumor and TAS. Several genes of antiviral immune response pathways such as HLA-B, HLA-C, HLA-DQB1 IFI6, IFITM3, CD74, and tumor suppressor genes EFEMP1 and EGR1, are upregulated in tumor and downregulated in TAS (Figure 3B, Supplementary Figure 5). Low expression levels of the MHC class II transporter and antigen presenting gene CD74 in TAS as compared to tumor of BA vs WA PCa patients may suggest a reduced tumor antigen presentation in TAS in BA patients, as class II MHC processing and regulation cannot properly occur in the absence of CD74 [44]. Additionally, several other genes associated with poor prognosis in other cancers were upregulated in TAS but downregulated in tumor of BA vs WA patients including oncogene SOX4 [26] and USP6 [27]. Data suggest a transcriptional regulatory effect of SOX4 on genes of multiple pathways that may play roles in prostate cancer progression [45]. Analysis of primary tumor samples have identified high expression of USP6 mainly in mesenchymal cancer including sarcomas [46].

The presence of both adaptive and innate immune cells has been identified within prostate TAS. However, variations in the immune cell density in tumor samples from different races are not well understood. We used CIBERSORT to estimate the fractions of 22 immune cell types in both tumor and TAS of PCa populations (Figure 3E) and found a high fraction of resting or immature NK cells in both BA and WA TAS of PCa patients. The immunosuppressive activities of immature NK cells in hematological malignancies have been reported [47]. In breast cancer worse disease-free survival (DFS) and overall survival (OS) rates were associated with higher cell fractions of M0 macrophage and resting NK cell fractions [48].

Interestingly, our analysis revealed differences in the representation of CD8 T-cells in TAS between BA and WA PCa patients (11% vs 6%, respectively). Although the association between high fractions of tumor-infiltrating CD8 T-cells and favorable prognosis have been reported in several tumors, in PCa the prognostic values of CD8 T-cells are unclear. Indeed, multiple studies suggest an association between poor clinical outcomes and shorter biochemical recurrence with higher proportions of both epithelial and stromal CD8 T-cells [49].

The tumor of BA compared to WA PCa patients show similarities in overall lymphocytic infiltration such as M1 macrophages and monocytes. However, our data suggest that there are fewer antigen-presenting cells (activated DCs) in BA vs WA PCa patients (Figure 3E). Activated mature DCs are important components of the innate immune response to tumor and essential targets in efforts to generate therapeutic immunity against cancer [50]. Lower fractions of activated DCs in the tumor of BA patients may therefore suggest a clinically unfavorable cytotoxic immune response to the tumor.

The small sample size of this investigation posed limitations on our ability to clinically match and analyze TAS data. Another limitation is that the TAS and tumor epithelial were drawn from two different cohorts. Furthermore, neither our TAS dataset nor the external dataset GSE54460 contains information on parameters that may affect gene expression and disease outcome, such as smoking status [51]. However, our exploratory transcriptome analysis represents an important step in understanding the underlying biology of prostate tumor epithelium and TAS of BA and WA patients. Our study indicates striking differences in immunoregulatory gene activities in TAS and tumor epithelium of BA compared to WA PCa patients. Subsequent studies will focus on whether these differences contribute to the worse prognosis of PCa in BA patients and whether therapeutic interventions can be developed that exploit these differences.

## MATERIALS AND METHODS

### Patients’ characteristics and RNA sequencing analysis of TAS of BA and WA PCa patients

To study gene expression differences in tumor adjacent stroma of BA vs WA PCa patients, samples from radical prostatectomies were obtained by informed consent using Institutional Review Board (IRB)-approved and HIPAA-compliant protocols at the University of California, Irvine (*n* = 11) and the Medical University of South Carolina (*n* = 9) (Supplementary Table 1). From Formalin Fixed Embedded (FFPEs) PCa tissues, multiple 20-micron thick tissue sections were generated and mounted on plastic microscope slides. The sites of tumor and tumor adjacent stroma are identified by superimposing the plastic slides on the marked H & E slide (Supplementary Table 1).

The FFPE RNA/DNA Purification Plus Kit (Cat # 54300, Norgen Biotek Corp) was used to isolate total RNA from nine BA and eleven WA PCa patient specimens. RNA quality and quantity were assessed using an Agilent 2100 Bioanalyzer and a Qubit fluorimeter, respectively. All samples exhibited a DV200 metric of over 30% of RNA with fragment sizes over 200 nucleotides, as previously described [52–54]. RNA expression profiling was performed using the Illumina Truseq RNA Access library preparation kit. The quality of libraries was assessed after PCR amplification using the Agilent 2100 Bioanalyzer.

Raw RNA-seq data were imported into the Strand NGS tool. Transcript quantification was performed using the DESeq normalization method (Strand NGS) followed by normalization to the mean of all samples. Pooled analysis was performed using the Audic Claverie (AC) test, which tests the differences between the two groups based on the assumption that sequence counts follow a Poisson distribution. The Benjamini-Hochberg correction was applied, and *p*_adj_ < 0.05 was used as the threshold for detection of differentially expressed genes.

### Transcriptome analysis of prostate tumor tissues from BA and WA patients

Raw RNA-seq data of tumor epithelium from 99 PCa patients was obtained from the GEO database accession GSE54460 [15], along with clinical data (Supplementary Table 2). Included in this study were 22 and 46 self-reported Black and White patients, respectively. Of the 99 patients, 31 had missing ancestry information. The GSE54460 prostatectomy samples were from three independent sites (Atlanta VA Medical Center, Sunnybrook Health Sciences Center at the University of Toronto, and Moffitt Cancer Center) [15]. After LASER analysis (see below), the following criteria for matching BA and WA were used for the GSE54460 dataset: A ratio of one BA to two WA in each category including biochemical relapse, age, Gleason sum score, follow-up time in months, and ancestry composition (80–100%). After matching for clinical parameters, sample site (i.e., VA only) and removing duplicates (*n* = 6), a total of 45 patients (15 BA and 30 WA) were eligible for further analysis (Supplementary Tables 2 and 3).

### Locating ancestry from sequence reads (LASER)

We used *Locating Ancestry from Sequence Reads* (LASER) software to verify the racial ancestry of the patients included in our study. LASER estimates individual ancestry by directly analyzing sequence reads without calling genotypes [18]. The program places each sample into a reference *Principal Component Analysis* (PCA) space constructed using 632,958 SNPs of reference individuals from all major geographical groups. We used the Human Genome Diversity Panel of LASER, which contains 1064 individuals from 51 populations worldwide from sub-Saharan Africa, North Africa, Europe, the Middle East, South/Central Asia, East Asia, Oceania, and the Americas [16, 17]. The estimated coordinates of the sequence samples from the reference individual then reflect the coordinates of known ancestral backgrounds [18], which can be used to determine the ancestry of experimental samples. The LASER analysis considers each person’s genome as having originated from K ancestral but unobserved populations whose contributions are described by K coefficients that sum to 1 for each individual [16]. We used the K-nearest neighbor (K = 10) to estimate the ancestry composition. Note that the individual ancestry proportion estimation is dependent on K and becomes more accurate with higher K-nearest neighborhoods.

### Molecular pathway analysis

Molecular pathway and functional analyses of statistically significantly differentially expressed genes of multiple experiments were analyzed using the Ingenuity Pathway analysis (IPA) package (QIAGEN Inc, USA) and the pathway analysis module in Strand NGS 3.1. We identified overrepresented canonical pathways and diseases/functions based on –log_10_ (*p*) >1.3 or *p* < 0.05.

### Identification of the immune microenvironment using CIBERSORT

*Cell-type Identification by Estimating Relative Subsets of RNA Transcripts* (CIBERSORT) [28] was used to characterize the immune cell type composition of each sample, which examined 22 immune cell types. Normalized quantified sequencing values were used as input to CIBERSORT. We calculated the relative immune fraction score (i.e., percentages of immune cells), which estimates the fraction of each immune cell type such that the sum of all fractions is equal to 1 (total leukocyte content) for a given mixture sample. We used *p* < 0.05 as the threshold for significance [28].

## SUPPLEMENTARY MATERIALS








