# Supplementary material for: RNA expression differences in prostate tumors and tumor-adjacent stroma between Black and White Americans
Source: Oncotarget. 2021 Jul 20;12(15):1457–69. doi: 10.18632/oncotarget.28024 (PMC8310667; doi:10.18632/oncotarget.28024)
Supplement: Supplementary file 1 [file oncotarget-12-1457-s001.pdf]

RNA expression differences in prostate tumors and tumor-adjacent stroma between Black and White Americans

SUPPLEMENTARY MATERIALS

Supplementary Dataset 1: RNA sequencing analysis of TAS of Black American (BA) and White American (WA) prostate cancer (PCa) patients. See Supplementary Dataset 1

Supplementary Table 1A: 3550 differentially transcribed genes in 7 BA prostate TAS cases versus 7 WA prostate TAS cases; corrected $p < 0.05$; FC ≥ 2.5 in both directions.

Supplementary Table 1B: Canonical pathways of 1706 significantly differentially downregulated transcribed genes in 7 BA prostate TAS cases versus 7 WA prostate TAS cases; corrected $p < 0.05$.

Supplementary Table 1C: Canonical pathways of 1844 significantly differentially upregulated transcribed genes in 7 BA prostate TAS cases versus 7 WA prostate TAS cases; corrected $p < 0.05$.

Supplementary Dataset 2: Analysis of published RNA sequencing data of prostate cancer tissue samples from BA and WA patients. See Supplementary Dataset 2

Supplementary Table 2A: 1408 differentially transcribed genes in 15 BA prostate tumor cases versus 30 WA prostate tumor cases; corrected $p < 0.05$; FC ≥ 1.5 in either direction.

Supplementary Table 2B: Canonical pathways of 932 significantly differentially upregulated transcribed genes in 15 BA prostate tumor cases versus 30 WA prostate tumor cases; corrected $p < 0.05$.

Supplementary Table 2C: Canonical pathways of 476 significantly differentially downregulated transcribed genes in 15 BA prostate tumor cases versus 30 WA prostate tumor cases; corrected $p < 0.05$.

Supplementary Table 2D: Diseases and functions associated with 932 significantly differentially upregulated transcribed genes in 15 BA prostate tumor cases versus 30 WA prostate tumor cases; corrected $p < 0.05$.

Supplementary Table 2E: Diseases and functions associated with 476 significantly differentially downregulated transcribed genes in 15 BA prostate tumor cases versus 30 WA prostate tumor cases; corrected $p < 0.05$.

Supplementary Table 2F: Gene network analysis of 476 significantly differentially downregulated transcribed genes in 15 BA prostate tumor cases versus 30 WA prostate tumor cases; corrected $p < 0.05$.

Supplementary Table 2G: Gene network analysis of 932 significantly differentially upregulated transcribed genes in 15 BA prostate tumor cases versus 30 WA prostate tumor cases; corrected $p < 0.05$.

Supplementary Dataset 3: Transcriptome analysis of BA versus WA PCa patients in tumor and TAS. See Supplementary Dataset 3

Supplementary Table 3A: 2500 differentially transcribed genes with the highest significance in 7 BA prostate TAS cases versus 7 WA prostate TAS cases; corrected $p < 0.05$.

Supplementary Table 3B: 2500 differentially transcribed genes with the highest significance in 15 BA prostate tumor cases versus 30 WA prostate tumor cases; corrected $p < 0.05$.

Supplementary Table 3C: Comparative pathway analysis on top differentially transcribed genes downregulated in BA versus WA patients between prostate TAS and tumors; corrected $p < 0.05$.

Supplementary Table 3D: Comparative pathway analysis on top differentially transcribed genes upregulated in BA versus WA patients between prostate TAS and tumors; corrected $p < 0.05$.

Supplementary Table 3E: Gene network analysis of 149 discordant genes in prostate TAS and tumors of BA versus WA patients; corrected $p < 0.05$.

Supplementary Table 3F: Gene network analysis of 94 concordant genes in prostate TAS and tumors of BA versus WA patients; corrected $p < 0.05$.

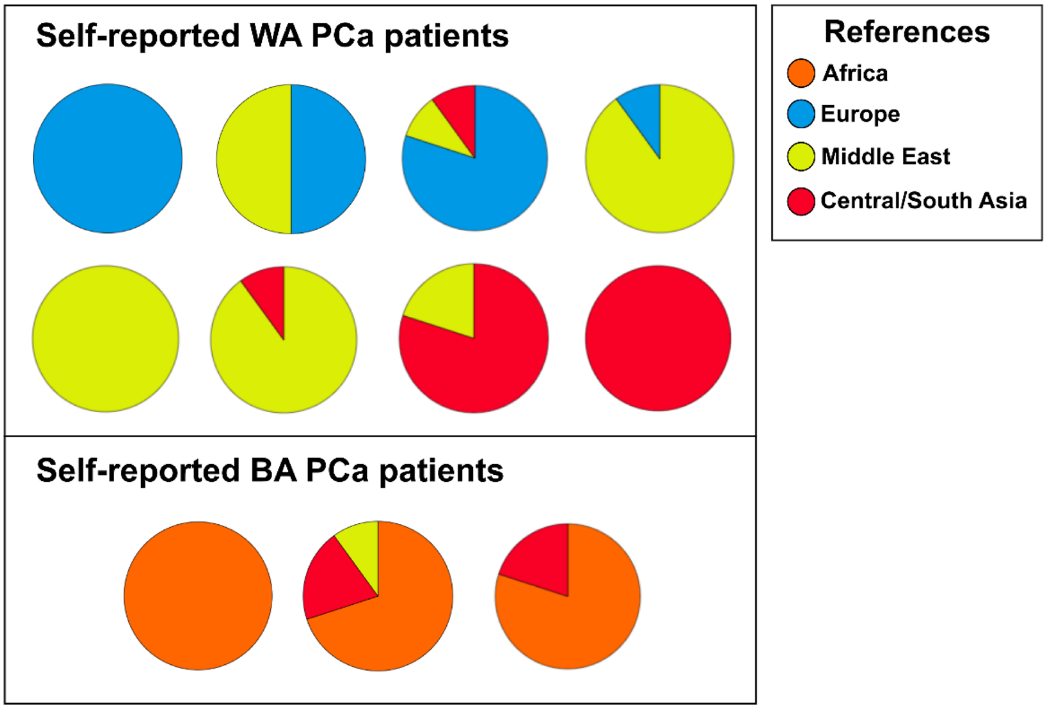

Supplementary Figure 1: Venn diagrams of the different ancestry compositions identified using LASER in 99 FFPE samples from BA and WA PCa patients (GSE54460).

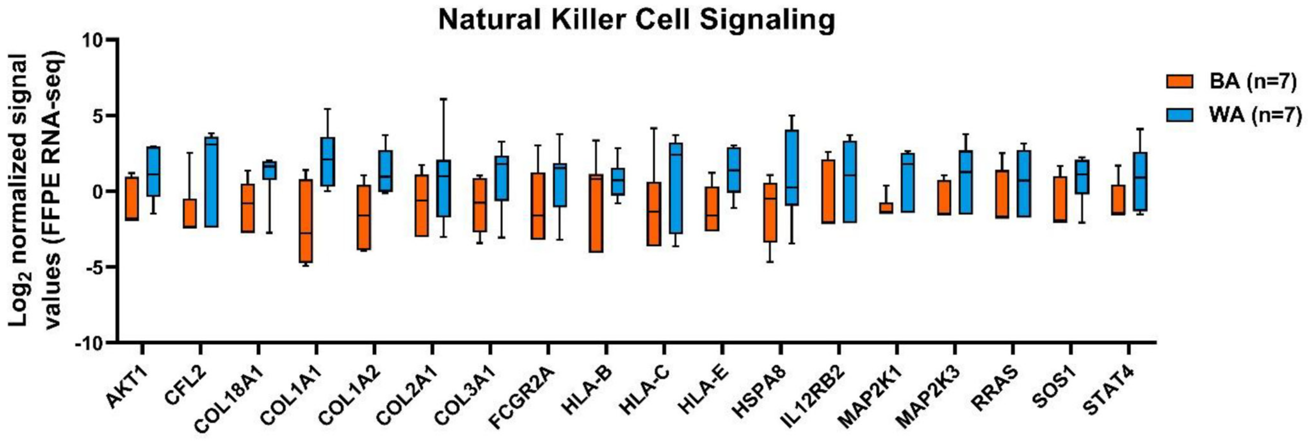

Supplementary Figure 2: Natural killer cell signaling genes significantly downregulated in the TAS of BA (n = 7) compared to WA (n = 7) prostate cancer samples. Y axis represents normalized $\log_2$ signal values (normalized reads) (p_{adj} <0.05). Each dot represents a patient.

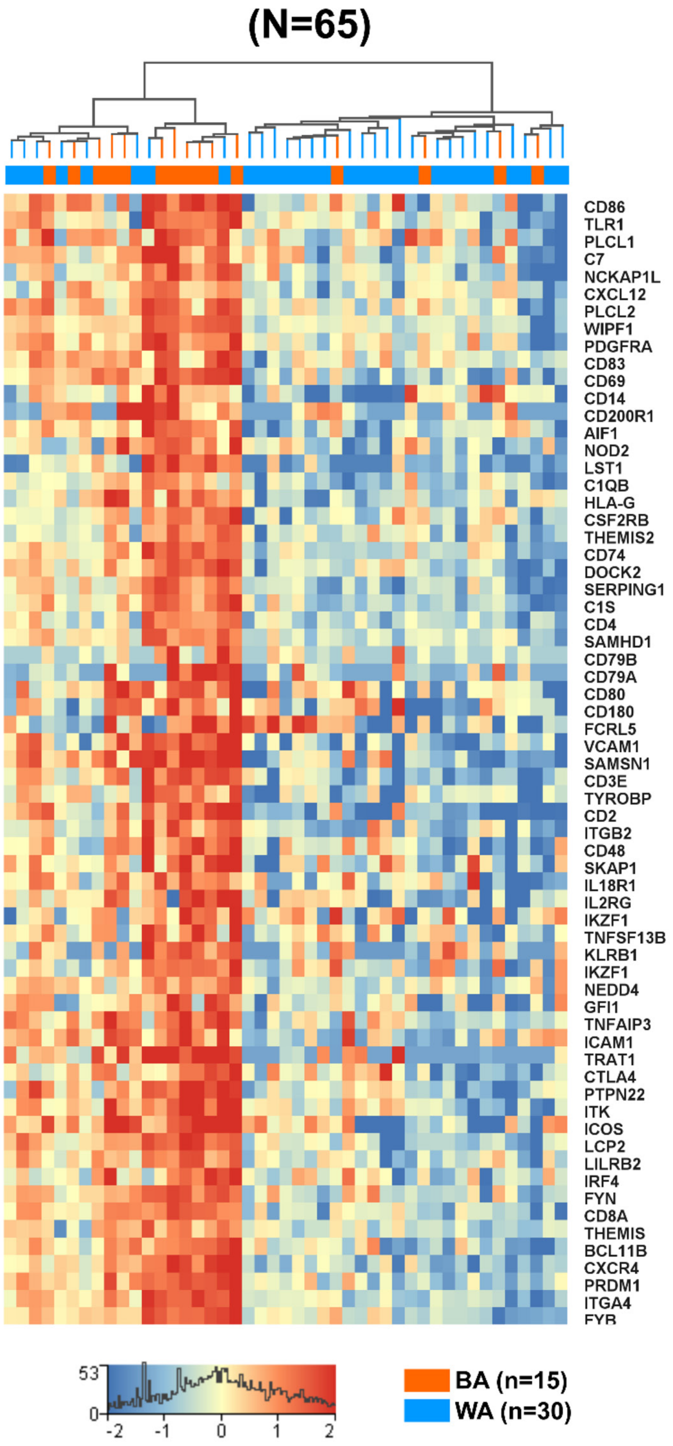

Supplementary Figure 3: Comparison of published regulation status of genes in BA vs WA PCa samples and our gene list. A study by Hardiman et al. from 10 self-reported BA and 17 self-reported WA PCa patients identified 242 upregulated genes [21].We identified 65 overlapping genes with our list of 1408 genes, at 98% concordance. The heat map shows the expression levels of those 65 overlapping concordant genes in 15 BA compared to 30 WA prostate cancer patients. Red depicts up- and blue depicts downregulation. N is the number of genes.

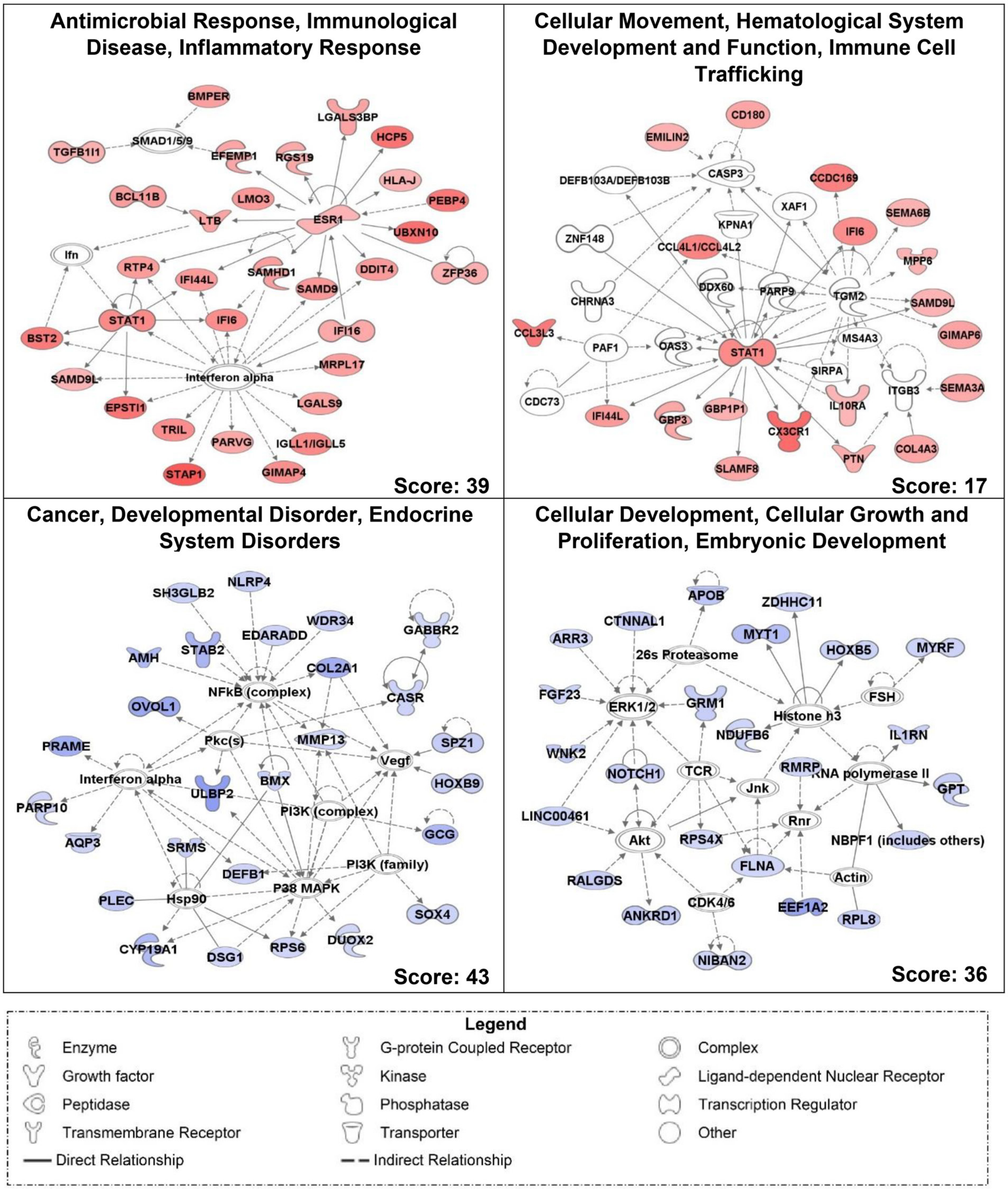

Supplementary Figure 4: Gene network analysis of statistically significant (corrected *p value* < 0.05) up- and downregulated genes in BA (*n* = 15) versus WA (*n* = 30) PCa samples. The networks shown are among those with the highest significance of connections between molecules in the network as indicated by their score. Blue nodes indicate downregulated gene expression and red nodes indicate upregulated gene expression. Darker shades of the nodes indicate higher regulation ratios. Dotted lines represent indirect interactions while solid lines represent direct interactions. White colored notes are in the pathway, but not in our dataset.

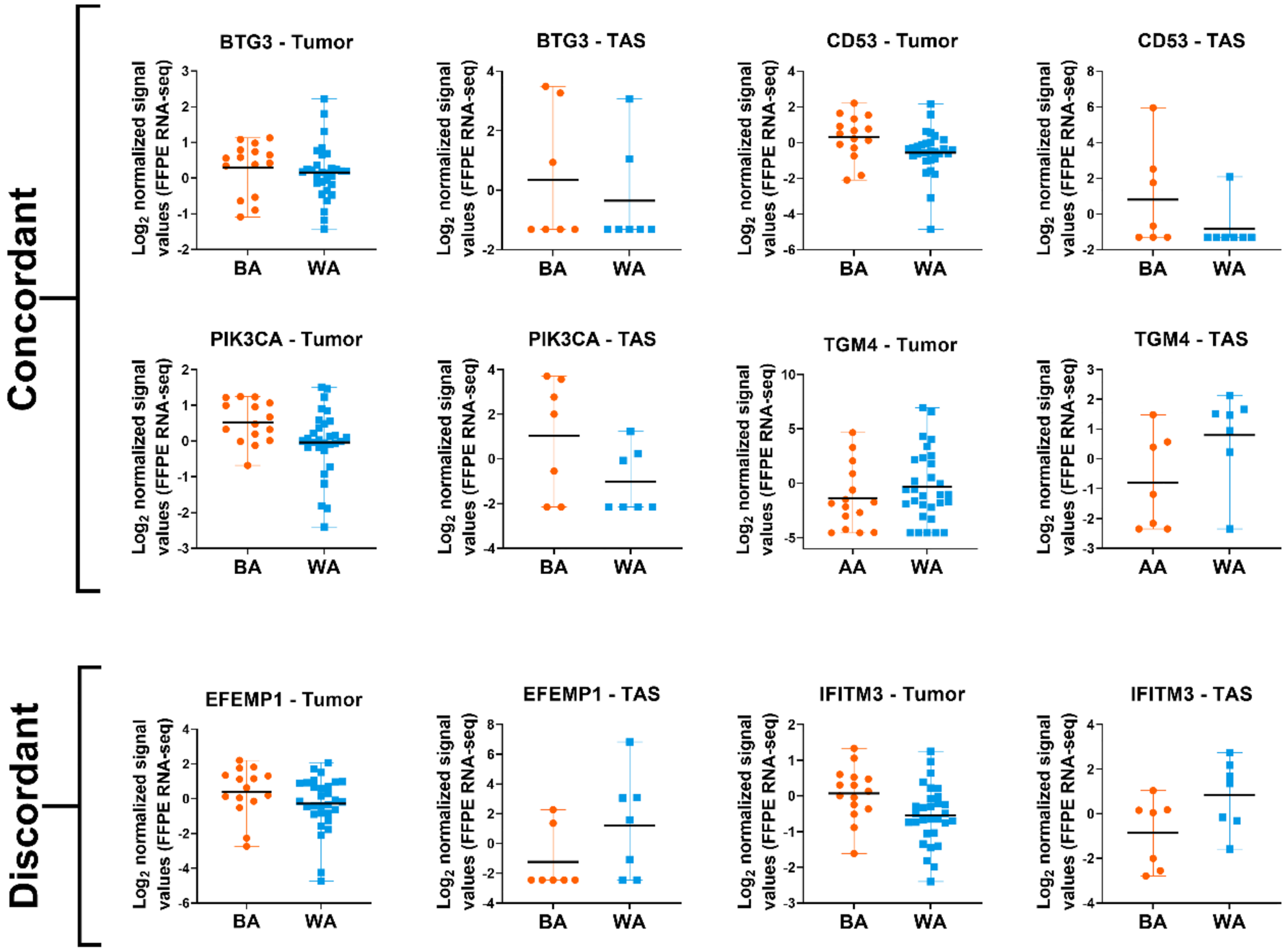

Supplementary Figure 5: Overlapping genes in tumor and TAS of BA versus WA PCa patients with concordant and discordant regulations.

Supplementary Table 1: Transcriptome analysis and clinical characteristics of TAS of self-reported BA (n = 9) and WA (n = 11) PCa patients

PCa-TAS	Reported Race	Location	Relapse Definition	Follow-up (months)	Gleason Score	Total Gleason Score	Given Age	Race Ratio	Race Breakdown	Number of Reads	Aligned Reads
TAS1***	Black	MUSC	R	24	3+5	8	60	7:2:1	Africa: C/S Asia: Middle East	58,353,208	9,146,862
TAS2***	Black	MUSC	N	59	3+4	7	61	10:0	Africa	17,275,140	9,540,061
TAS3***	Black	MUSC	R	1	3+4	7	66	10:0	Africa	100,431,162	28,995,535
TAS4***	Black	MUSC	R	73	3+3	6	60	6:2:2	Africa: C/S Asia: Middle East	14,218,314	8,651,441
TAS5***	Black	UCI	R	11	4+5	9	57	10:0	Africa	46,529,804	22,448,895
TAS6***	Black	MUSC	R	23	4+5	9	64	7:2:1	Africa: C/S Asia: Middle East	13,857,852	5,685,592
TAS7***	Black	MUSC	N	36	3+4	7	62	5:3:2	Africa: C/S Asia: Middle East	9,567,576	3,693,855
TAS8	Black	MUSC	R	89	3+4	7	56	6:4	Middle East: C/S Asia	34,398,988	4,202,710
TAS9	Black	UCI	N	13	3+4	7	66	6:4	Middle East: C/S Asia	175,188,492	65,452,830
TAS10***	White	UCI	R	95	4+4	8	62	10:0	Middle East	8,702,216	5,104,814
TAS11***	White	UCI	N	82	3+4	7	66	10:0	Middle East	60,372,832	35,386,525
TAS12***	White	UCI	R	24	3+4	7	53	8:1:1	Europe: Middle East: C/S Asia	19,391,098	11,692,864
TAS13***	White	UCI	R	19	4+4	8	43	10:0	Europe	3,800,718	1,053,081
TAS14***	White	UCI	R	16	4+5	9	63	10:0	Europe	18,000,216	6,707,433
TAS15***	White	UCI	R	17	4+4	8	68	8:2	Europe: Middle East	49,658,208	17,847,446
TAS16***	White	UCI	R	22	4+3	7	66	10:0	Europe	5,539,412	1,309,553
TAS17	White	UCI	N	61	3+3	6	60	10:0	C/S Asia	31,396,544	17,660,670
TAS18	White	UCI	R	90	3+4	7	63	9:1	C/S Asia: Europe	26,390,916	8,587,454
TAS19	White	UCI	R	2	4+5	9	59	7:3	C/S Asia: America	14,017,038	3,781,150
TAS20	White	UCI	R	15	4+5	9	67	9:1	C/S Asia: Europe	5,513,162	1,008,259

Abbreviations: TAS: Tumor adjacent stroma; PCa: Prostate cancer; UCI: University of California, Irvine; MUSC: Medical University of South Carolina; C/S: Central/South; R: Relapse; N: Non-relapse. (*) denotes patients selected for gene expression analysis.

Supplementary Table 2: Clinical characteristics and geographical ancestry of GSE54460 PCa patients

PCa-Tumor	Reported Race	Location	Given Age	BCR (months)	Gleason Score	Total Gleason Score	Follow-up (months)	Race Ratio	Race Breakdown
BA1*	Black	VA	63	NA	5+3	8	59.5	10:0	Africa
BA2*	Black	VA	62	NA	3+4	7	61.5	10:0	Africa
BA3*	Black	VA	69	NA	3+4	7	67.9	10:0	Africa
BA4*	Black	VA	70	NA	3+4	7	106.6	10:0	Africa
BA5*	Black	VA	47	NA	3+4	7	69.8	10:0	Africa
BA6*	Black	VA	56	NA	3+3	6	51.5	10:0	Africa
BA7*	Black	VA	62	NA	3+4	7	71.9	10:0	Africa
BA8*	Black	VA	54	NA	3+4	7	73.8	10:0	Africa
BA9*	Black	VA	50	NA	3+4	7	77.3	10:0	Africa
BA10*	Black	VA	53	1.6	3+4	7	1.6	10:0	Africa
BA11*	Black	VA	66	26.6	4+3	7	26.6	10:0	Africa
BA12*	Black	VA	55	0.7	4+5	9	0.7	10:0	Africa
BA13*	Black	VA	58	60.5	3+4	7	60.5	10:0	Africa
BA14*	Black	VA	52	26	3+4	7	26	10:0	Africa
BA15*	Black	VA	62	2.2	4+4	8	2.2	9:1	Africa: C/S Asia
BA16	Black	VA	69	NA	3+4	7	69.5	8:2	Africa: C/S Asia
BA17	Black	VA	56	NA	3+4	7	68	8:2	Africa: C/S Asia
BA18	Black	VA	56	NA	3+4	7	118.3	9:1	Africa: C/S Asia
BA19	Black	VA	50	NA	3+4	7	96.6	10:0	Africa
BA20	Black	VA	57	NA	4+3	7	89.3	7:2:1	Africa: C/S Asia: Middle East
BA21	Black	VA	60	26.6	4+3	7	26.6	7:2:1	Africa: C/S Asia: Middle East
BA22	Black	VA	58	101.1	3+4	7	101.1	8:2	Africa: C/S Asia
BA23	NA	MCC	43	NA	3+4	7	50	10:0	Africa
BA24	NA	UTP	55	0	3+4	7	50	10:0	Africa
BA25	NA	UTP	71	0	3+4	7	54	10:0	Africa
BA26	NA	UTP	51	0	4+3	7	100	10:0	Africa
BA27	NA	VA	59	36	4+4	8	36	10:0	Africa
WA1*	White	VA	62	NA	4+3	7	62.4	10:0	Europe
WA2*	White	VA	52	NA	3+4	7	50.4	10:0	Europe
WA3*	White	VA	51	NA	3+3	6	64.2	10:0	Europe
WA4*	White	VA	64	NA	4+4	8	171.1	10:0	Europe
WA5*	White	VA	69	NA	3+4	7	48.6	10:0	Europe
WA6*	White	VA	70	NA	3+4	7	116.5	10:0	Europe
WA7*	White	VA	65	NA	3+4	7	148.2	10:0	Europe
WA8*	White	VA	67	NA	3+4	7	116.9	10:0	Europe
WA9*	White	VA	64	NA	3+4	7	155.1	10:0	Europe
WA10*	White	VA	61	NA	4+3	7	31.8	10:0	Europe
WA11*	White	VA	50	NA	3+4	7	100.2	8:1:1	Europe: Middle East: C/S Asia
WA12*	White	VA	54	NA	3+4	7	95.9	10:0	Europe
WA13*	White	VA	61	NA	3+4	7	83	9:1	Middle East: C/S Asia
WA14*	White	VA	54	NA	3+4	7	64.3	10:0	Europe
WA15*	White	VA	55	NA	3+4	7	82.3	10:0	Middle East
WA16*	White	VA	66	NA	4+3	7	55.6	10:0	Middle East

WA17[*]	White	VA	62	NA	3+4	7	34.7	10:0	Europe
WA18[*]	White	VA	65	NA	3+3	6	63.4	10:0	Middle East
WA19[*]	White	VA	58	3.6	4+3	7	3.6	10:0	Europe
WA20[*]	White	VA	64	0.8	4+4	8	0.8	10:0	Europe
WA21[*]	White	VA	67	3.6	3+4	7	3.6	10:0	Europe
WA22[*]	White	VA	52	1	4+4	8	1	10:0	Europe
WA23[*]	White	VA	67	95	3+4	7	95	10:0	Europe
WA24[*]	White	VA	67	18.3	4+3	7	18.3	10:0	Europe
WA25[*]	White	VA	65	1.6	4+4	8	1.6	10:0	Europe
WA26[*]	White	VA	62	3.5	3+4	7	3.5	10:0	Europe
WA27[*]	White	VA	67	11.8	3+4	7	11.8	9:1	Middle East: Europe
WA28[*]	White	VA	62	48.9	4+3	7	48.9	10:0	Middle East
WA29[*]	White	VA	59	2.8	4+3	7	2.8	9:1	Europe: Middle East
WA30[*]	White	VA	57	39.9	3+4	7	39.9	10:0	Europe
WA31	White	VA	68	NA	3+4	7	63.6	7:3	Europe: Middle East
WA32	White	VA	65	NA	3+4	7	160.3	8:2	Europe: Middle East
WA33	White	VA	72	NA	3+4	7	97.9	8:2	Europe: Middle East
WA34	White	VA	65	NA	3+4	7	76.8	8:2	Middle East: Europe
WA35	White	MCC	78	NA	3+4	7	119	10:0	Europe
WA36	White	MCC	61	NA	3+3	6	114	10:0	Europe
WA37	White	MCC	63	NA	3+3	6	87	7:3	Middle East: Europe
WA38	White	MCC	57	NA	3+4	7	69	10:0	Europe
WA39	White	MCC	54	NA	3+4	7	57	10:0	Europe
WA40	White	MCC	66	26.5	3+3	6	104.05	10:0	Europe
WA41	White	MCC	61	22.13	3+3	6	74.24	10:0	Middle East
WA42	White	MCC	60	154.22	3+2	5	180.56	10:0	Europe
WA43	White	UTP	58	18	3+4	7	43	10:0	Europe
WA44	White	UTP	72	0	4+4	8	112	10:0	Europe
WA45	NA	MCC	58	NA	3+4	7	98	10:0	Europe
WA46	NA	UTP	57	NA	4+3	7	72	10:0	Europe
WA47	NA	UTP	66	NA	4+4	8	85	10:0	Europe
WA48	NA	UTP	50	NA	3+3	6	63	10:0	Europe
WA49	NA	UTP	69	NA	3+4	7	82	10:0	Europe
WA50	NA	UTP	71	38.2	5+4	9	38	10:0	Europe
WA51	NA	UTP	50	23	3+4	7	68	10:0	Europe
WA52	NA	UTP	68	27.6	4+3	7	82	10:0	Middle East
WA53	NA	UTP	55	0	3+4	7	49	8:2	Europe: Middle East
WA54	NA	UTP	65	8	3+4	7	128	10:0	Europe
WA55	NA	UTP	62	21.4	3+3	6	116	10:0	Europe
WA56	NA	UTP	58	0	3+4	7	102	10:0	Europe
WA57	NA	UTP	67	0	4+5	9	26	5:5	Europe: Middle East
WA58	NA	UTP	70	0	4+4	8	35	10:0	Europe
WA59	NA	UTP	59	0	4+3	7	88	10:0	Europe
WA60	NA	UTP	53	43.4	5+4	9	75	10:0	Middle East
WA61	NA	UTP	60	29.8	3+4	7	43	10:0	Europe
WA62	NA	UTP	60	48.5	3+4	7	49	7:3	Europe: Middle East

WA63	NA	UTP	65	27.6	4+3	7	101	10:0	Europe
WA64	NA	UTP	74	18.2	3+4	7	79	10:0	Europe
WA65	NA	UTP	66	49.5	3+4	7	125	10:0	Europe
WA66	NA	UTP	57	27.5	3+4	7	112	9:1	Europe: Middle East
WA67	NA	UTP	66	0	4+3	7	72	10:0	Europe
WA68	NA	UTP	72	9.6	4+3	7	36	5:5	Europe: Middle East
AS1	White	VA	64	NA	3+3	6	97.3	10:0	C/S Asia
AS2	White	VA	64	NA	4+3	7	96.1	8:2	C/S Asia: Middle East
AS3	NA	UTP	64	21.6	4+3	7	89	10:0	C/S Asia
AS4	NA	UTP	58	29.9	4+3	7	61	10:0	C/S Asia

Abbreviations: BA: Black American; WA: White American; AS: Asian; VA: Atlanta Veterans Administration Medical Center; MCC: Moffitt Cancer Center; UTP: University of Toronto Sunnybrook Health Sciences Center; C/S: Central/South; BCR: biochemical relapse; NA: Not applicable. (*) denotes patients selected for gene expression analysis.

Supplementary Table 3: Summary of clinically matched BA (*n* = 15) and WA (*n* = 30) FFPE PCa tumor-enriched samples (GSE54460)

	n	No BCR: BCR	Average Age	Gleason Score 6	Gleason Score 7	Gleason Score 8	Gleason Score 9	Average Follow-Up (Months)	80-90% Ancestry	100% Ancestry
BA	15	9:6	59	1	11	2	1	50	1	14
WA	30	18:12	61	2	24	4	0	59	4	26

Abbreviations: BA: Black American; WA: White American; BCR: biochemical relapse.
